# Environmental variables associated with Arbuscular Mycorrhizal Fungi diversity in Ohio soybean agricultural systems

**DOI:** 10.1007/s00572-026-01321-z

**Published:** 2026-10-07

**Authors:** Samaneh Tajik, Florence Breuillin-Sessoms, Timothy Frey, Jelmer W. Poelstra, Horacio D. Lopez-Nicora, Maria Soledad Benitez Ponce

**Affiliations:** 1 Ohio State University Extension and Department of Food, Agricultural and Biological Engineering, The Ohio State University, Columbus, OH USA; 2 Department of Horticulture and Crop Science, The Ohio State University, Wooster, OH USA; 3 Department of Plant Pathology, The Ohio State University, Wooster, OH USA; 4 The Ohio State University, Department of Plant Pathology, Columbus, OH USA; 5 CFAES Bioinformatics Core, The Ohio State University, Wooster, OH USA

**Keywords:** Arbuscular mycorrhizal fungi, Soybean, Agricultural systems, Soil properties, AML1/AML2

## Abstract

Arbuscular mycorrhizal fungi (AMF) play a crucial role in crop productivity and agroecosystem processes. However, the composition of soil AMF communities and the factors associated with their diversity and distribution in Ohio remain poorly understood. We profiled both soil AMF and physico-chemical characteristics in 18 soybean fields across agricultural regions in Ohio. AMF sequences were recovered through amplicon sequencing of the 18S ribosomal gene coupled with PacBio technology. We detected 14 genera belonging to six families of the Glomeromycota. The family *Glomeraceae* accounted for 39% of the AMF reads recovered, while the families Gigasporaceae and Archaeosporaceae were rare (detected in < 25% of samples). At the genus level, *Entrophospora*, *Nanoglomus*, *Paraglomus*, and *Funneliformis* were widespread, contrasting with rare taxa such as *Gigaspora*, *Dominikia*, and *Archaeospora*. For the studied soil parameters, only phosphorus was significantly associated with AMF community composition. These findings provide a baseline of AMF communities in Ohio soybean fields and highlight abiotic components influencing these communities. Future research should expand sampling to other regions of the state and evaluate root colonization and evaluation of AMF impacts on soybean yields in Ohio.

**Supplementary Information:**

The online version contains supplementary material available at https://doi.org/10.1007/s00572-026-01321-z.

## Introduction

Arbuscular mycorrhizal fungi (AMF) are soil microorganisms able to form symbiotic associations with the roots of most agricultural plants. In agricultural systems, AMF have been shown to contribute to nutrient mobilization and uptake, and increased plant tolerance to biotic and abiotic stresses (Wen et al. 2023). Mycorrhizal associations can potentially increase crop productivity, reduce fertilizer application and support sustainable agriculture. In soybean (*Glycine max*), the crop occupying the largest planted area in Ohio agricultural systems, AMF inoculation results in increased growth and yield (Igiehon et al. 2021). Previous work has shown that soybean roots associate with multiple AMF genera (Igiehon et al. 2021; Wen et al. 2023), and that these associations can be influenced by management practices. For instance, in Kojima et al. (2014), *Glomus* sp. was among the most frequently detected taxa in soybean roots, and its detection frequency varied across soil types and land use histories. In addition, AMF community composition shifts with soybean phenological growth stages. Regardless, the rhizosphere is typically dominated by *Glomus* morphotypes rather than *Acaulospora* and *Gigaspora* (Buade et al. 2021), and AMF diversity is influenced by broader environmental conditions rather than previous crops alone (Kojima et al. 2014).

The landscape-scale distribution of AMF communities in soils and plant roots is driven by soil properties, the local environment, and host species occurrence (Hazard et al. 2013). Soil pH (Xu et al. 2017), soil texture (Xu et al. 2017), nutrient availability, and organic matter (Smith and Read 2008) affect AMF community composition. For instance, Moebius-Clune et al. (2013), Goldmann et al. (2020) and Aker et al. (2022) identified soil texture as an important factor associated with AMF landscape distribution. Further, Moebius-Clune et al. (2013) proposed that the influence of soil texture on AMF community structure is related to soil moisture dynamics and nutrient availability. Biotic variables such as host genotype, plant diversity, and interactions with other soil organisms can also influence AMF community composition and function. For example, microorganisms that alter root structure, carbon allocation, and nutrient availability can affect the plant’s ability to support mycorrhizal symbiosis (Poll et al. 2007). Conversely, AMF can mitigate the effects of abiotic and biotic stressors, including soilborne plant pathogens, by improving nutrient uptake and by priming plant defense responses (Sharma et al. 2026).

Although AMF are recognized as key contributors to soil health and crop productivity, their distribution across locations and taxonomic diversity in Ohio agricultural systems remain poorly understood. In this study, we characterized the diversity of native AMF in soils under agricultural production, identified the dominant taxa, and summarized their relationship with abiotic soil components across Ohio. Evaluating native AMF diversity and distribution across various agricultural soil types will provide the baseline necessary to maximize the benefits of native or amended AMF-soybean interactions. We hypothesized that AMF communities across soil types and soybean production regions would vary, with unique taxa recovered in different regions. We further quantified AMF relationships with soil parameters (pH, available nutrients, organic matter).

## Materials and methods

### Soil samples

A total of 28 soil samples were initially selected from a larger soil survey conducted to assess fungal communities associated with SCN in Ohio (Medina López et al. 2024). Of these, the 18 soil samples (Table 1) in which Glomeromycota reads were recovered were used for further analyses (Supplementary Materials and Methods, Supplemental Table 1). Briefly, bulk soils were collected from agricultural fields under soybean and corn rotation using a 2.5 cm diameter soil probe at approximately 15 cm depth. A composite soil sample was obtained from 15 soil cores per location, and DNA extraction was performed on a freeze-dried subsample. Further details on soil sampling (including sampling date and management characteristics), measurement of soil parameters, and soil DNA extraction procedures are described in Medina López et al. (2024), Supplementary Materials and Methods and Supplemental Table 1. Available phosphorus (P) was specifically measured on these soils using the Bray P1 extraction method (Bray and Kurtz 1945) at the Ohio State University’s STAR lab (https://u.osu.edu/starlab/).

**Table 1:** Soil properties measured across the studied soil samples

Sample ID^a^	County	Soil order^b^	pH^a^	TEC^a^	OM^a^	PP
				meq/100 g	%	ppm
FS4	Fulton	Entisol	5.8	18.3	3.36	59.9
FS5	Fulton	Entisol	5.2	14.9	2.39	17.7
FS11	Ross	Inceptisol	6.2	22.6	4.31	34.3
FS14	Ross	Alfisol	6.3	26.9	4.14	21.8
FS19	Allen	Mollisol	6.4	11.7	2.69	40.5
FS38	Miami	Alfisol	7.7	17.4	2.71	39.1
FS39	Miami	Alfisol	6.2	11.6	2.5	23
FS41	Tuscarawas	Alfisol	5.7	11.9	2.33	21.4
FS43	Tuscarawas	Alfisol	6.9	22.9	2.68	43.9
FS44	Van Wert	Alfisol	6	13.2	3.04	62
FS47	Van Wert	Alfisol	5.5	12.4	3.21	28.7
FS48	Wyandot	Alfisol	6.5	15.5	3.41	20.2
FS51	Wyandot	Alfisol	6.5	11.7	3.24	138.7
FS54	Darke	Alfisol	7.2	13.73	3.05	157.3
FS58	Knox	Alfisol	6.6	22.9	4.69	48.4
FS62	Knox	Alfisol	7.7	23.4	3.2	58.8
FS63	Union	Mollisol	6.5	11.6	2.6	41.4
FS65	Union	Alfisol	6.3	13.3	3.47	47.4

^a^ Soil ID, soil pH, Total exchange capacity (TEC), organic matter (OM) were previously determined in Medina López et al. (2024)

^b^ Soil order was determined by the geographical location of the samples using Web Soil Survey data compiled by the National Cooperative Soil Survey and USDA-NRCS

### AMF amplicon sequencing

For the selected samples, DNA libraries for AMF community analysis through amplicon sequencing with Pacific Biosciences technology (PacBio, Menlo Park, CA, USA) were prepared using a two-step PCR process. First, sequences targeting the small subunit rRNA gene were amplified using AMF-specific primers AML1 (5′-ATCAACTTTCGATGGTAGGATAGA-3′) and AML2 (5′-GAACCCAAACACTTTGGTTTCC-3′) (Lee et al. 2008) tailed with universal sequences (5’ blocked, F - /5AmMC6/GCAGTCGAACATGTAGCTGACTCAGGTCAC-primer-3’, R–5’/5AmMC6/TGGATCACTTGTGCAAGCATCACATCGTAG-primer-3’) for the addition of the barcode primers. PCR conditions recommended by PacBio were followed with annealing temperatures at 61 °C for AML1/2 (Pacific Biosciences of California 2020), and the KAPA HiFi HotStart polymerase (Roche Sequencing Solutions 2021). The product from these reactions was then used to attach PacBio barcode primers (PacBio Barcoded Universal F/R Primers Plate-96v2) through PCR with conditions as recommended by the manufacturer, 20 cycles and a 64 °C annealing temperature. Barcoded samples were then pooled using best practices for equimolar pooling. Libraries were concentrated and cleaned using AMPure beads (Beckman Coulter, Brea, CA, USA). Clean AMF amplicon library concentrations were checked using a Qubit (ThermoFisher, Oakwood, CA) before submission to the University of Louisville for PacBio sequencing. The remaining library preparation and sequencing, including the attachment of SMRTbell adaptors, was carried out at the University of Louisville Sequencing Technology Center. Additional information is available in the Supplementary Materials and Methods. Raw sequences are available at the NCBI SRA, BioProject PRJNA1445323.

### Bioinformatic processing

PacBio long reads were preprocessed to remove primers and adapters using cutadapt v. 5.0 (Martin 2011). The DADA2 pipeline v.1.26 (Callahan et al. 2016) in R v.4.4.0 (R Core Team 2024) was used to infer amplicon sequencing variants (ASVs), adapted for long-read sequences (Callahan et al. 2019). This adaptation allows quality scores up to 93 and sequence lengths up to 3,200 bp. DADA2’s *filterAndTrim* function (options: maxEE = 2, minLen = 500, maxLen = 3200) was used for filtering and trimming, the *learnErrors* function (options: multi = TRUE, verbose = TRUE) for error rate prediction, the *dada* function for denoising into ASVs, and the *removeBimeraDenovo* function (options: minFoldParentOverAbundance = 4.5, multi = TRUE, verbose = TRUE) for chimera detection and removal. ASVs were classified using DADA2’s *assignTaxonomy* (Callahan et al. 2016) function with a bootstrap value threshold of 80 against a slightly modified version of the Eukaryome database (Tedersoo et al. 2024). Specifically, sequences from the Eukaryome FASTA file assigned to the kingdoms Mitochondrion and Plastid were removed, as described in Supplementary Materials and Methods. All code used for bioinformatic processing and data analysis can be found in the GitHub repository https://github.com/jelmerp/amf-metabarcoding.

### Data analysis

Only sequences assigned to the phylum *Glomeromycota*, which contains all known AMF taxa (Schüßler et al. 2001), and samples that met quality control criteria were used for downstream analyses (see Supplementary Materials and Methods). Relative abundances were calculated as frequencies within and across samples. To facilitate qualitative comparisons of AMF community structure across the locations studied, we calculated occurrence frequencies within and across samples. Within each individual sample, genera were either classified as dominant (≥ 20% of total sequence reads), intermediate (5–20% of reads), or rare (< 5% of reads). For across-sample comparisons, the percentage of presence of each genus across all samples was calculated using a per-sample detection threshold of one read. Across samples, genera were classified as dominant (detected in ≥ 75% of samples), intermediate (detected in 25%–75% of samples), and rare (detected in < 25% of samples) (Robinson-Boyer et al. 2009; Zhang et al. 2021; Pascoal et al. 2025).

AMF alpha diversity was calculated using the *phyloseq* package v.1.46.0 in R (McMurdie and Holmes 2013). Shannon (H′) and Simpson (D) diversity indices were calculated at the ASV level with the function *estimate_richness*. To evaluate variation in AMF community composition among samples, a weighted UniFrac distance matrix was calculated. This approach incorporates both ASV phylogenetic relationships and relative abundances (Lozupone and Knight 2005). Permutational multivariate analysis of variance (PERMANOVA; *adonis2* function in the vegan package) was used to test the effects of soil properties on AMF community composition. Given the limited sample size, all models were kept simple and included only main effects without interactions. To further assess the relationship between environmental variables and AMF genera, a distance-based PCoA analysis (vegan package) was conducted using the same distance matrix. Site scores and environmental vectors were extracted to visualize the strength and direction of associations between AMF communities and soil properties. Spearman’s correlation coefficients were also calculated to examine relationships between individual AMF genera and soil properties (*stats* package in R).

## Results

### Variability of soil characteristics across Ohio counties

Table 1 summarizes soil properties (pH, TEC, OM, P) of the 18 studied fields sampled across ten counties in Ohio and corresponding soil orders (Medina López et al. 2024). Soil pH ranged from acidic (5.2 in FS5) to slightly alkaline (7.7 in FS62 and FS38). Total exchange capacity ranged from 11.6 meq/100 g (FS39) to 26.9 meq/100 g (FS14), while OM content varied from 2.33% (FS41) to 4.69% (FS58). Soil available P levels showed the greatest variation, from 17.7 ppm (low available P, FS5) to 157.3 ppm (high available P, FS54), consistent with observed Ohio soil fertility patterns (Mercer and Butler 2018). Soil macronutrients and micronutrients for the 18 tested soils (Supplemental Table 1) did not indicate nutrient deficiency. The observed differences in soil properties provided a representative pedogenic environment relevant to AMF community structure for Ohio agronomic crop production.

### AMF metabarcoding sequencing and community composition

A total of 162,764 reads were assigned to Glomeromycota across the selected 18 samples (per-sample mean: 9,042; range 402–22,490, Table 2), corresponding to 549 distinct ASVs. Details on parameters used for sample selection, filtering, and original sequencing outputs are described in the Supplementary Materials and Methods. Supplemental Table 3 summarizes the number of reads observed per sample before and after various filtering steps. Shannon diversity index estimates ranged from 1.6 (FS65) to 4.0 (FS19), while Simpson indices varied from 0.80 to 0.98 for the same samples, indicating high community evenness across sites.

**Table 2:** AMF read counts and calculated diversity indices across soil samples and Ohio counties

Sample ID	County	AMF reads	Shannon diversity (ASV)	Simpson diversity (ASV)
FS11	Ross	4,928	3.3	0.94
FS14	Ross	3,413	2.1	0.86
FS19	Allen	13,948	4	0.98
FS38	Miami	13,173	3.7	0.97
FS39	Miami	6,794	3.8	0.97
FS4	Fulton	6,274	3.6	0.96
FS41	Tuscarawas	5,379	3.7	0.97
FS43	Tuscarawas	4,969	3.6	0.96
FS44	Van Wert	9,167	3.7	0.96
FS47	Van Wert	22,490	3.8	0.96
FS48	Wyandot	19,685	3	0.87
FS5	Fulton	9,313	3.7	0.96
FS51	Wyandot	2,597	3	0.94
FS54	Darke	966	2.6	0.91
FS58	Knox	9,458	3.4	0.95
FS62	Knox	19,403	3.9	0.97
FS63	Union	10,405	3.1	0.91
FS65	Union	402	1.6	0.8

**Fig. 1:**
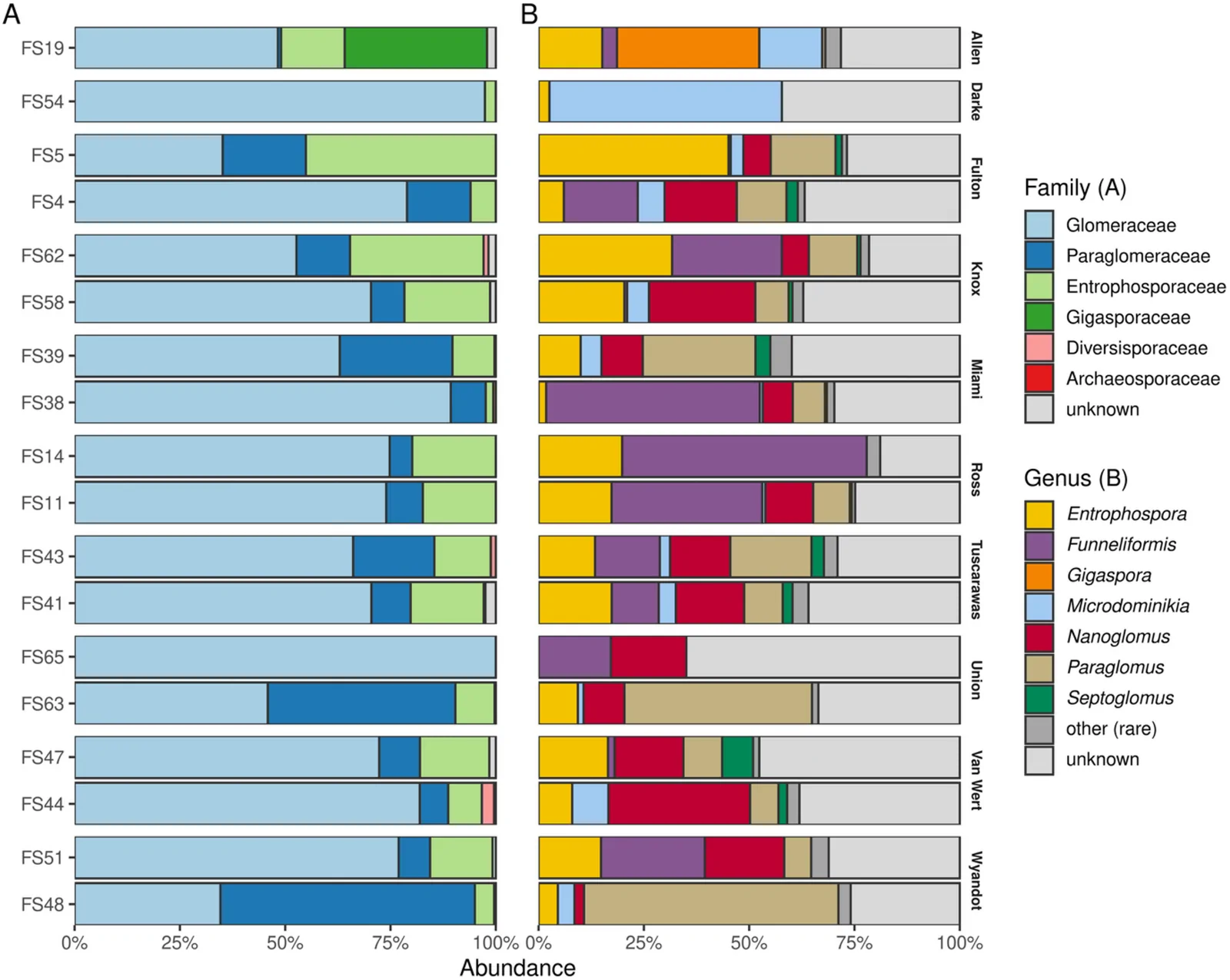
Relative abundances of arbuscular mycorrhizal fungi (AMF) recovered from 18 sampled soils across Ohio at the family (**A**) and genus (**B**) level. “Unknown” represents ASVs classified as Glomeromycota but unclassified at the family or genus level, respectively. At the genus level, “Other (rare)” corresponds to genera with relative abundance < 1%

Six AMF families (Glomeraceae, Paraglomeraceae, Entrophosporaceae, Diversisporaceae, Gigasporaceae, and Archaeosporaceae) were detected in samples from the 18 fields studied across ten counties (Fig. 1). Glomeraceae was the dominant family, occurring in all samples and representing more than 50% of AMF reads in 15 of the 18 samples. The only exceptions were samples FS48 and FS5, where Glomeraceae accounted for approximately 35% of total Glomeromycota reads. Paraglomeraceae was detected in 83% of samples and was generally observed as a subdominant family contributing 5–20% of reads in most samples. Paraglomeraceae exceeded Glomeraceae in sample FS48, where it represented 60% of reads. Similarly, Entrophosporaceae was observed in 89% of the samples, and accounted for 10–20% of reads; Entrophosporaceae was dominant in sample FS5, where it represented 45% of reads. Among the remaining families, Diversisporaceae was detected in 39% of samples at low relative abundance (< 5%). Gigasporaceae and Archaeosporaceae were rare families that were each detected in only one sample, with 34% and less than 1% of AMF reads, respectively.

Genus-level results supported the patterns observed at the family level and the main genera detected in most samples belonged to the dominant and subdominant families. A total of 14 genera were detected across all samples, including *Paraglomus*, *Funneliformis*, *Entrophospora*, *Gigaspora*, *Nanoglomus*, *Microdominikia*, *Septoglomus*, *Diversispora*, *Sclerocystis*, *Rhizoglomus*, *Epigeocarpum*, *Dominikia*, *Archaeospora*, and an unknown Glomeraceae genus (Glomeraceae_gen17) (Table 3; Fig. 1). Several genera were widely recovered from the studied soils; *Entrophospora* (87%), *Nanoglomus* (83%), *Paraglomus* (83%) and *Funneliformis* (72%), showed the highest prevalence among all samples. In contrast, *Gigaspora* was detected in only one sample (6% prevalence), although at a high relative abundance within that sample (47%, Fig. 1). Approximately 52,000 AMF reads were not classified to genus.

**Table 3:** Summary of the AMF genera recovered across 18 Ohio field sites

Family	Genus	Total reads (*n*)	Relative abundance (%)	Prevalence (*n*, %)	Max relative abundance (%)
Glomeraceae	*Funneliformis*	19,617	15.39	13 (72%)	72
	*Nanoglomus*	17,831	13.99	15 (83%)	54
	*Microdominikia*	6,291	4.91	12 (67%)	96
	*Septoglomus*	2,977	2.34	11 (61%)	14
	*Sclerocystis*	1,204	0.94	7 (39%)	7
	*Rhizoglomus*	1,278	1.00	7 (39%)	4
	*Epigeocarpum*	294	0.23	3 (17%)	2
	Unknown genus (Glomeraceae_gen17)	238	0.19	3 (17%)	4
	*Dominikia*	113	0.09	1 (6%)	1
Paraglomeraceae	*Paraglomus*	29,339	23.02	15 (83%)	81
Entrophosporaceae	*Entrophospora*	25,531	19.94	16 (89%)	62
Diversisporaceae	*Diversispora*	695	0.55	7 (39%)	5
Gigasporaceae	*Gigaspora*	4,713	3.70	1 (6%)	47
Archaeosporaceae	*Archaeospora*	61	0.05	1 (6%)	< 1

### pH effect on AMF community composition

**Fig. 2:**
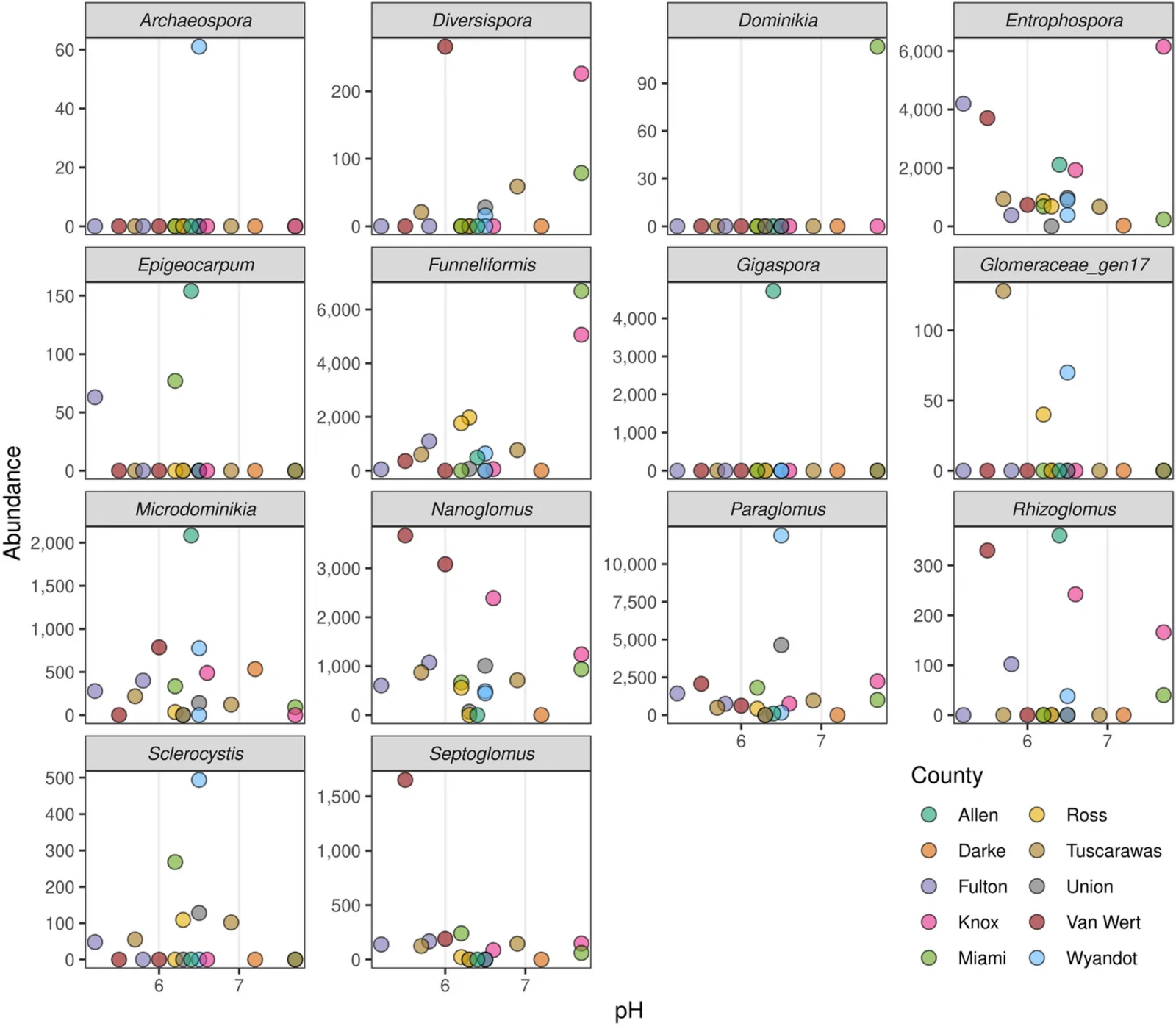
AMF genus-level abundance across soil pH in the counties studied. The x‑axis in every panel is soil pH, the y‑axis is read abundance, and each county has a distinct color

AMF community composition varied across the observed soil pH range as seen in Fig. 2. Most AMF genera were observed within the pH range of 5.5–6.5. Several genera, including *Paraglomus*, *Nanoglomus*, *Microdominikia*, *Septoglomus*, and *Rhizoglomus*, were more prevalent in slightly acidic to neutral soils. Genera that were observed across a wide pH range included *Funneliformis*, *Entrophospora*, and *Diversispora*, with highest abundances at slightly alkaline pH (7.7). *Entrophospora* was also detected at high abundance in acidic soil (pH 5.2). In contrast, rare genera (*Gigaspora*, *Archaeospora*) were detected at pH 6.4–6.5.

### Variation in AMF community composition among sampled fields

**Fig. 3:**
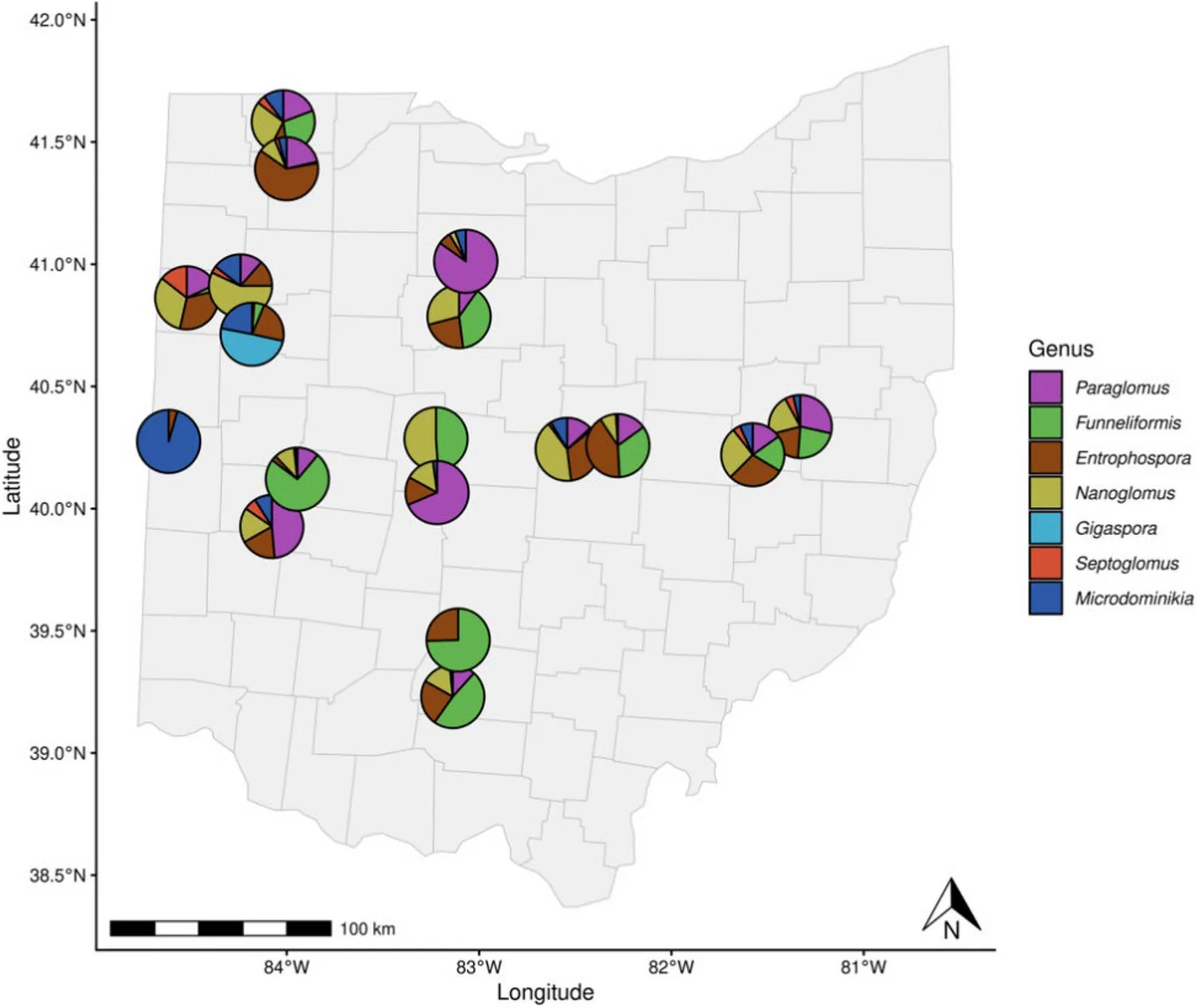
Distribution of arbuscular mycorrhizal fungi (AMF) genera abundance across sampled Ohio counties. Pie chart colors indicate AMF genera

AMF community composition varied among sampled locations. (Fig. 3). The 18 studied samples were collected across ten Ohio counties and showed a wide range of fertility levels (Table 1; Supplemental Table 1). Most fields were dominated by one or more of four genera: *Paraglomus*, *Funneliformis*, *Entrophospora*, and *Nanoglomus* (Fig. 3). *Funneliformis* was predominant in five fields (FS11, FS14, FS38, FS51, and FS62) accounting for up to 72% of recovered reads. In contrast, *Paraglomus* was most abundant in FS39, FS48, and FS63, with its highest relative abundance in FS48 (81%). *Entrophospora* dominated FS5, while *Nanoglomus* was particularly abundant in FS44, FS58, and FS65, and *Microdominikia* in FS54. A second group of genera, including *Microdominikia*, *Septoglomus*, *Rhizoglomus*, *Diversispora*, and *Sclerocystis*, was observed at moderate abundance across the sampled fields but generally contributed less than 15% of recovered Glomeromycota reads. The latter genera rarely dominated individual samples and were commonly detected together with members of the Glomeraceae, Paraglomeraceae, and Entrophosporaceae.

The genera *Gigaspora*,* Archaeospora* and *Dominikia* showed limited distribution across sites, each detected in a single field. Although *Gigaspora* was detected in only one field, it represented 47% of the observed AMF reads at that location. In contrast, *Archaeospora* and *Dominikia* were detected at low abundance (< 1%).

### Principal coordinates analysis (PCoA) of soil properties and AMF community composition

**Fig. 4:**
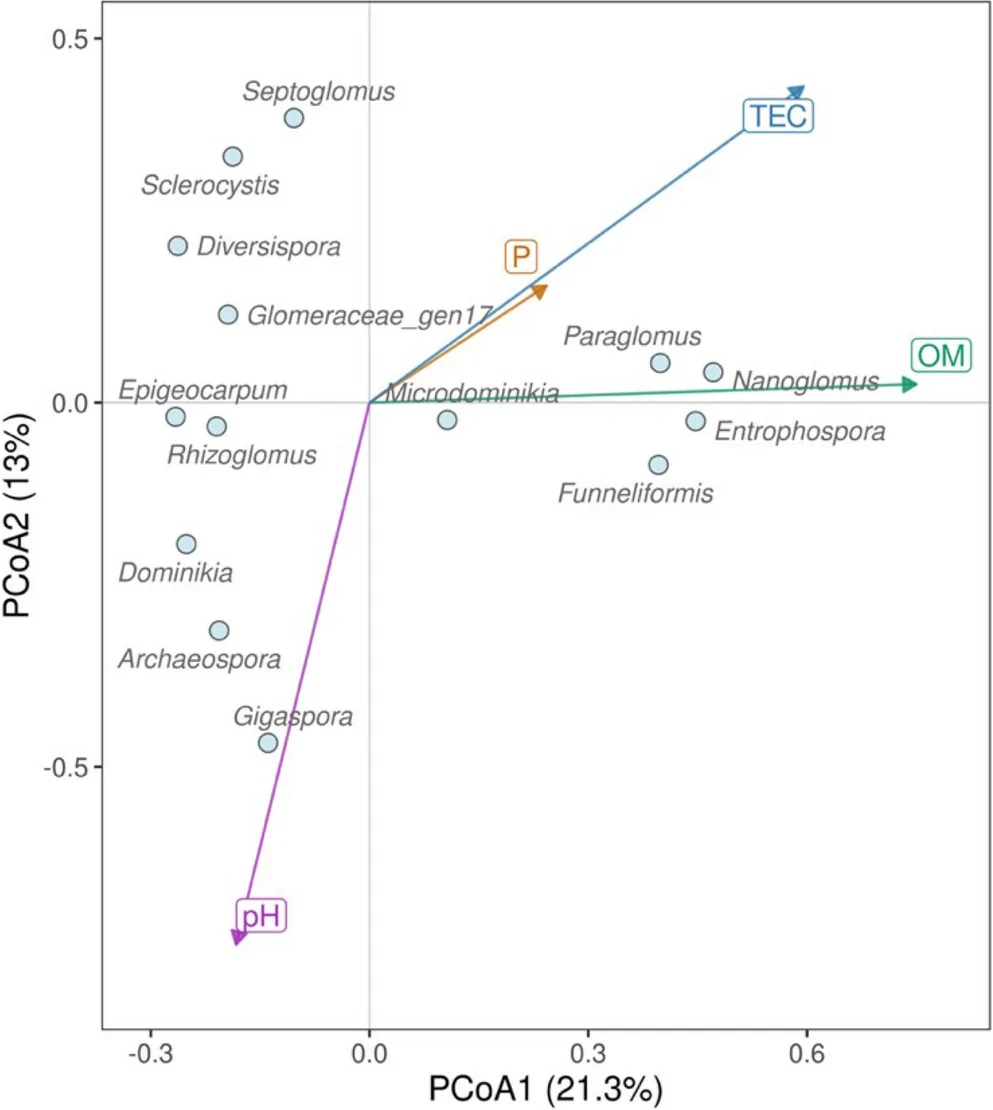
Taxa-centric Principal Coordinates Analysis (PCoA) of arbuscular mycorrhizal fungi (AMF) genera and soil properties (pH, OM, TEC, P; colored vectors)

PCoA analysis based on weighted UniFrac distances indicated variation in AMF genera among field samples. The first two axes explained 34% of the variance (Fig. 4). Soil OM and pH vectors correlated with the first and second axis, respectively, with P and TEC clustering in the same region as OM. *Microdominikia*, *Paraglomus*, *Nanoglomus*, *Entrophospora*, and *Funneliformis* were associated with OM, TEC, and available P, whereas *Gigaspora*, *Archaeospora*, and *Dominikia* were associated with pH. Based on PERMANOVA (Table 4), phosphorus was significantly associated with AMF community composition across samples (R² = 0.222, *P* = 0.027; Supplemental Fig. 4).

**Table 4:** PERMANOVA analysis of soil properties associated with AMF community composition based on UniFrac distances

Variable	Df	*R*²	F	*P*-value
pH	1	0.01	0.30	0.89
OM	1	0.03	0.57	0.57
P	1	0.22	4.72	0.03*
TEC	1	0.03	0.55	0.66
Residual	13	0.61	-	-
Total	17	1.00	-	-

Soil pH (pH), organic matter (OM), available phosphorus (P), and total exchange capacity (TEC). * *P*-value < 0.05

## Discussion

### AMF communities in Ohio soils

Using amplicon metabarcoding of Glomeromycota-specific markers, coupled with long-read sequencing with PacBio, we characterized AMF communities in Ohio soybean production fields. The combination of published ribosomal AMF markers (Lee et al. 2008), high-fidelity polymerase, long read PacBio amplicon sequencing, DADA2 (Callahan et al. 2016) and the Eukaryome database (Tedersoo et al. 2024) allowed us to improve the detection of AMF taxa in soils. These communities varied across sampled locations, with different families and genera dominating at different sites throughout Ohio soils. DADA2 provides accurate error correction and superior ASV recovery compared with traditional OTU-based clustering methods (Callahan et al. 2016; Babis et al. 2024). In a benchmarking study, DADA2 output most closely matched the expected (mock) community composition, especially when considering alpha and beta diversity measures (Fares et al. 2025), making it one of the most accurate pipelines for fungal and AMF metabarcoding analyses. This method allowed us to classify most Glomeromycota ASVs to the genus level and to distinguish some genera like *Paraglomus* (Paraglomeraceae) and *Entrophospora* (Entrophosporaceae) from Glomeraceae and other families (Delavaux et al. 2022; Błaszkowski et al. 2022).

Glomeraceae was the most abundant AMF family recovered in this study (mean relative abundance: 39%). Nine Glomeraceae genera were detected: *Funneliformis*,* Nanoglomus*,* Microdominikia*,* Septoglomus*,* Rhizoglomus*,* Sclerocystis*, *Epigeocarpum*,* Dominikia*, and an unclassified genus (Glomeraceae_gen17), some of which were observed across soils that differed in their soil chemical properties. These genera occupy a broad range of environmental conditions, showing Glomeraceae dominance across diverse environments. The predominance of Glomeraceae observed in this study is consistent with previous reports from agricultural systems, where members of this family frequently occur under managed and disturbed conditions, and across varied soil conditions (Krishnamoorthy et al. 2015; Xu et al. 2017; Ducousso-Détrez et al. 2022). Species of the Glomeraceae can rapidly colonize roots and persist under agricultural disturbance (Schalamuk and Cabello 2010), including fertilization, variable nutrient conditions (Lin et al. 2012), and annual cropping systems or rotations (Xu et al. 2017). *Funneliformis* and *Nanoglomus* are also reported to maintain strong host colonization under stress conditions (Meng et al. 2026).

In contrast, families such as Paraglomeraceae and Entrophosporaceae were each represented by a single genus (*Paraglomus* and *Entrophospora*, respectively) and occurred at a lower relative abundance (23% and 20%, respectively) compared to the Glomeraceae family. These families were present in more than 90% of samples, suggesting ecological relevance in these agricultural fields. However, in this study, root colonization was not evaluated.

*Paraglomus* showed high prevalence across samples and was the most abundant genus in several field samples. However, no consistent association with the measured environmental variables was observed. This observation aligns with van der Heijden et al. (2015), who described that AMF families like Paraglomeraceae exhibit broader environmental tolerances. Although Glomeraceae has been reported to persist and perform well in disturbed soils, previous studies have reported increased relative abundance of *Paraglomus* under environmental conditions where other AMF taxa are less dominant (Säle et al. 2021; MacColl et al. 2024). *Paraglomus’* widespread but subdominant presence might suggest ecological roles that differ from those of Glomeraceae (van der Heijden et al. 2015).

*Entrophospora* was the most prevalent genus detected and observed across a wide range of our field samples and study conditions. *Entrophospora* was more common in samples with higher AMF diversity, although this pattern was not uniform in all sites. Its high abundance and highest prevalence indicate that *Entrophospora* is widespread in Ohio, suggesting a broad environmental tolerance, which is consistent with previous reports (Błaszkowski et al. 2022; Silva et al. 2025) .

The families of Glomeraceae, Paraglomeraceae and Entrophosporaceae are the core of the recovered AMF families in the studied soils, possibly resulting from similar responses to environmental gradients. The coexistence of a dominant family, such as Glomeraceae with more specialized groups like Paraglomeraceae and Entrophosporaceae may indicate that each community member contributes differently to plant colonization and performance (Säle et al. 2021). While Glomeraceae likely provides a stable, broadly adapted component of the AMF community to agricultural production, the consistent presence of these two other families suggests a potential role in supporting plant or fungal community resilience. This is also supported by the variation in Shannon diversity values with consistently high Simpson indices, which indicates that while overall diversity varied among samples, dominant genera remained relatively stable across samples.

The low prevalence of Gigasporaceae, Diversisporaceae, and Archaeosporaceae is consistent with reports that these groups occur less frequently in agricultural soils. However, because Gigasporaceae and Archaeosporaceae were detected in only one field, and given the limited sampling of this study, it was not possible to draw conclusions regarding the environmental factors influencing their occurrence. In prior research, however, Gigasporaceae was observed to perform well in undisturbed soils (Schalamuk and Cabello 2010), and their presence was positively correlated with soil micronutrients such as zinc and heavy metals like cadmium (Krishnamoorthy et al. 2015). Diversisporaceae species are often associated with distinct soil environments and can be found in agricultural systems (MacColl et al. 2024). Archaeosporaceae are known for low detectability and highly specialized environmental requirements (Lee et al. 2008; Säle et al. 2021), which may contribute to their infrequent detection in agricultural surveys. Despite the low abundance and low frequency of these families (Gigasporaceae, Diversisporaceae and Archaeosporaceae) in this study, it is not possible to discount their potential to colonize agricultural hosts and contribute to host and/or soil health. It is also possible that the total read counts recovered in this study reflect not only biological differences but also technical variation across samples introduced by methodological bias (Shaffer et al. 2025).

### Soil chemical properties impact Ohio AMF communities

The variation in pH, TEC, OM, and P observed in the studied fields is consistent with statewide patterns reported in the Baseline Assessment of Soil Health in Ohio (Culman et al. 2022). Soil properties correlate with native soil AMF (Avio et al. 2020), as observed in the PCoA analysis. The PCoA showed that soil properties, including pH, OM, TEC, and available P, were associated with different AMF genera. This finding agrees with earlier reports demonstrating that soil abiotic variables shape AMF community composition in agricultural systems (Xu et al. 2017; Avio et al. 2020). Several dominant genera, including *Funneliformis*, *Nanoglomus*, *Paraglomus*, and *Entrophospora*, were observed across a broad range of soil conditions, although their relative abundances varied among fields.

*Funneliformis* showed its highest relative abundance in soils with high pH (7.7, FS38) and TEC (26.9, FS14), suggesting that the combination of higher pH and greater available nutrients provides a buffered and chemically stable soil environment favorable to this genus. Furthermore, *Funneliformis* displayed a positive correlation with TEC (Spearman *r* = 0.48, *P* < 0.05), supporting the idea that this genus tends to perform well in soils with sufficient plant nutrient availability and moisture (Avio et al. 2020). Similarly, *Nanoglomus* was most abundant in several soils with higher available P, showing a positive correlation (Spearman, *r* = 0.53, *P* < 0.05). A previous study demonstrated that *Nanoglomus* reads were higher in soils with greater available P (Corazon-Guivin et al. 2024). A possible mechanism behind this observation is that in agricultural soils with high P, plants reduce their mycorrhizal P dependence, which can lead to a fast-colonizing strategy and lower carbon demand by *Nanoglomus*, while other taxa decline (Corazon-Guivin et al. 2024). This pattern was directly demonstrated by Corazon-Guivin et al. (2023) who showed *Nanoglomus* persistence and fast colonization in fertilized agricultural conditions.

Soil properties directly and indirectly shape the chemical environment surrounding roots (Xu et al. 2017) and so determine which AMF taxa can establish, persist, and compete within a specific soil. Variability in pH, OM, and nutrient availability could create distinct ecological filters that selectively favor some of the genera while limiting others and structuring the distribution and abundance of AMF between the Ohio field sites. In our study, OM, TEC, and available P were oriented in a similar direction in the PCoA plot, suggesting a positive relationship among these soil variables and some AMF genera. Also, this finding is consistent with prior research suggesting that soil properties, like P, may be a primary factor associated with variation in AMF community composition among agricultural fields. However, some samples with similar soil properties showed distinct AMF communities, indicating that additional environmental or management factors not measured in this study may contribute to AMF community composition. These findings align with previous research showing that AMF communities are shaped by both soil properties and crop history (Van Der Gast et al. 2011) and emphasize the complexity of AMF distribution in agricultural landscapes.

## Conclusion

Our findings suggest that AMF communities varied among the sampled Ohio soybean fields and were associated with local environmental and soil conditions. Glomeraceae, Paraglomeraceae, and Entrophosporaceae were broadly represented under the soil conditions and cropping systems sampled. *Paraglomus*,* Funneliformis*,* Entrophospora*, and *Nanoglomus* were the most widespread genera, whereas *Archaeospora* and *Gigaspora* occurred at low frequency. Although dominant genera were detected across a range of soil conditions, their prevalence varied among fields, suggesting that multiple environmental factors influence AMF community composition. Available soil P was the only soil variable significantly associated with AMF community composition. OM, TEC, and available P formed a cluster of environmental variables that were positioned opposite to soil pH. Genera such as *Paraglomus*, *Nanoglomus*, *Entrophospora*, *Microdominikia*, and *Funneliformis* were associated with the OM, TEC, and P gradient. These findings suggest that variation in AMF community composition across Ohio soybean fields is associated primarily with soil P availability, while broader trends in AMF genus distribution may also reflect underlying soil environmental gradients.

## Supplementary Information

Supplementary Material 1.

## Data Availability

Raw sequencing data reported in this paper has been deposited in NCBI Sequence read archives under BioProject number: PRJNA1445323.
