# Supplementary material for: Environmental variables associated with Arbuscular Mycorrhizal Fungi diversity in Ohio soybean agricultural systems
Source: Mycorrhiza. 2026 Oct 7;36(5):78. doi: 10.1007/s00572-026-01321-z (PMC13642480; doi:10.1007/s00572-026-01321-z)
Supplement: Supplementary file 1 [file 572_2026_1321_MOESM1_ESM.pdf]

Environmental variables associated with Arbuscular Mycorrhizal Fungi diversity in Ohio soybean agricultural systems

Samaneh Tajik[1,2], Florence Breuillin-Sessoms[2], Timothy Frey[3], Jelmer W. Poelstra[3,4], Horacio D. Lopez-Nicora[5], Maria Soledad Benitez Ponce[3*]

[1]The Ohio State University, Ohio State University Extension and Department of Food, Agricultural and Biological Engineering, Columbus, Ohio, United States

[2]The Ohio State University, Department of Horticulture and Crop Science, Wooster, Ohio, United States

[3]The Ohio State University, Department of Plant Pathology, Wooster, Ohio, United States

[4]The Ohio State University, CFAES Bioinformatics Core, Wooster, Ohio, United States

[5]The Ohio State University, Department of Plant Pathology, Columbus, Ohio, United States

ORCID iD

Samaneh Tajik: https://orcid.org/0000-0002-5896-0114

Florence Breuillin-Sessoms: https://orcid.org/0000-0002-9009-0841

Jelmer Poelstra: https://orcid.org/0000-0002-3514-7462

Timothy Frey: https://orcid.org/0000-0003-2623-7834

Horacio D. Lopez-Nicora: https://orcid.org/0000-0002-9672-9589

Maria Soledad Benitez Ponce: https://orcid.org/0000-0003-2314-1052

*Corresponding author, email: benitezponce.1@osu.edu

Supplementary Material

Supplemental Material and Methods

Soil sample selection and AML1/AML2 amplicon sequencing

A total of 28 soil samples were selected from a larger survey that was conducted to assess fungal communities associated with the soybean cyst nematode (SCN) in Ohio (Medina López et al., 2024). Soils were collected from agricultural productions fields under soybean and corn rotation during the growing season of 2021. Briefly, soil sampling followed recommendations for SCN detection (Dorrance et al 2018), with a total of 15 2.5 cm diameter by 15 cm deep soil cores collected from up to eight hectares. A composite sample per eight-hectare field was obtained by homogenization and sieving (2mm). A sub-sample was used for DNA extraction after freeze drying to standardize soil volume (Medina López et al., 2024). DNA libraries for AM fungal specific amplicon sequencing and amplicon sequencing variant (ASV) detection were performed as described in the Methodology section. The 30 samples (28 soils plus 2 controls) for AML1/AML2 were multiplexed with other experimental samples to be run in a single PacBio smart cell. Three databases were evaluated for taxonomic classification of ASV based on the description below. Supplemental Table 1summarizes sampling information for the final 18 samples considered in the analysis.

Taxonomic database selection

Using NCBI blastn (from the command-line BLAST+ program v. 2.16.0), we compared the performance of three taxonomic databases for our dataset: the "18S_fungal_sequences" BLAST database (downloaded on 2025-02-15), the MaarjAM SSU database (version 2021; Opik et al 2010), and the Eukaryome database (General Eukaryote SSU release version 1.9.3; Tedersoo et al 2024). To download the BLAST database, we used the BLAST+ command "update_blastdb.pl 18S_fungal_sequences". The MaarjAM database was downloaded from

https://maarjam.ut.ee/resources/maarjam_database_SSU.fasta.2021.zip. The MaarjAM database FASTA file was preprocessed for usage with blastn by removing the 120 duplicate entries that it contained with SeqKit v. 2.8.2 and editing the headers to only retain the NCBI accession numbers. Furthermore, an NCBI taxonomic ID to NCBI accession number lookup file was downloaded from NCBI (https://ftp.ncbi.nlm.nih.gov/pub/taxonomy/accession2taxid/nucl_gb.accession2taxid.gz) on 2025-02-22 and edited to only contain taxonomic ID and accession number columns without a header line. Finally, a BLAST database was created using the BLAST+ “makeblastdb” command with the edited MaarjAM FASTA file and the taxonomic lookup file as inputs. The Eukaryome database was downloaded from https://sisu.ut.ee/wp-content/uploads/sites/643/General_EUK_SSU_v1.9.3.zip, and its FASTA file was used to create a BLAST database using the BLAST+ “makeblastdb” command.

The FASTA file with the recovered ASV sequences after DADA processing, was independently BLASTed against all three databases using the blastn command with an E-value threshold of 1x10-6. The BLAST output was sorted by E-value, then by bitscore, and then by percent identity and for each query, only the top 5 hits per query were retained. For the NCBI and MaarjAM databases, which use NCBI taxonomic IDs, taxonomic classification (Kingdom|Phylum|Order etc) for each hit was added to the BLAST output using taxonkit v. 0.18.0.

Comparisons of the output showed that the Eukaryome database had by far the highest-quality BLAST hits, while the MaarJAM database performed worst (Supp. Fig. 1 and Supp. Fig. 2). We therefore opted to use the Eukaryome database to assign taxonomy to the recovered ASV.

Eukaryome classification performance evaluation and selection and removal of mitochondrion and plastid sequences

We compared the performance of different parameters and algorithms for taxonomic classification using the Eukaryome database. 1) the “assignTaxonomy” function from the DADA2 (v. 1.34.0) R package with two different bootstrap value thresholds: the default of 50 and a more restrictive threshold of 80, 2) Qiime2’s “feature-classifier” command (subcommands “fit-classifier-naive-bayes” followed by “classify-sklearn”), and 3) Vsearch’s “sintax” command (with confidence threshold “–sintax_cutoff 0.8”). As performance metrics, we used the proportion of ASV assigned taxonomy at various taxonomic levels, and concordance with high-quality BLAST hits (defined as those with at least 97% identity and at least 90% query/ASV coverage). Soil samples and positive control samples were used for this analysis. DADA2 had the highest assignment rates and highest BLAST concordance and was therefore chosen as the method for final taxonomic assignments.

However, we noticed that among assignments that differed from high-quality BLAST hits at high taxonomic levels, many were assigned to the "kingdom Mitochondrion", and this was particularly prevalent with the DADA2 classifier. Further investigation showed that in all such cases, the best Mitochondrion BLAST hit was short (below 30% ASV coverage) and with not particularly high percent identity (around 85-90%). These statistics were below the performance of fungal BLAST hits to these ASV, many of which had >97% identity and >90% ASV coverage. Even within the short sections that aligned to Mitochondrial sequences, percent identity was consistently lower than to the best fungal hit. Therefore, Mitochondrion sequences were removed from the database. Subsequently, dozens of ASV that had been assigned to Mitochondrion earlier were assigned to Plastid after the removal of Mitochondrion sequences, even though no good

Plastid BLAST hits were found, like with Mitochondrion. Therefore, Plastid sequences were also removed from the database. DADA2 taxonomic assignments with a bootstrap threshold of 80 had higher concordance with high-quality BLAST hits and only slightly fewer total assignments, and this bootstrap value was therefore used for final taxonomic assignments.

Sequencing summary and sample filtering

For the 28 soil samples and controls sequenced, 302,770 reads and 1,658 ASV were recovered after quality filtering, trimming, and DADA2 processing with the selected pipeline described above. Of these ASV, using the Eukaryome database, 90.3% were assigned a taxonomic label at any level, 72.3% to at the family level, 53.92% to the genus level, and 28.17% to the species level (see Supplementary Fig. 3). Blast analysis of the ASV revealed that the Eukaryome data base provided a higher percentage of positive hits compared to the commonly used Maarjam (Opik et al 2010) and NCBI 18S_fungal sequence (Supplemental Fig. 1 and 2). After taxomomic classification with Eukaryome, 639 ASV, were assigned to the Glomeromycota, with 8 soil samples removed due to no or low Glomeromycota read counts (<30). Two additional soil samples were removed due to incomplete data. Supplementary Table 3 summarizes sequenced samples, number of reads in different steps of the pipeline, including final AM reads.

Supplemental Tables

Supplemental Table 1. Sampling time and field characteristics at the time of sampling.

Sample ID[a]	County	Sampling date	Current crop	Crop history information [b]
FS11	Ross	April 2021 [b]	Fallow	NA
FS14	Ross	April 2021 [b]	Fallow	NA
FS19	Allen	July 24, 2021	Soybean	Soybean/Corn rotation
FS38	Miami	August 27, 2021	Soybean	Soybean/Corn rotation, No-till
FS39	Miami	August 27, 2021	Soybean	Soybean/Corn rotation, No-till
FS4	Fulton	July 6, 2021	Soybean	NA
FS41	Tuscarawas	Summer 2021[b]	NA [c]	NA
FS43	Tuscarawas	Summer 2021[b]	NA	NA
FS44	Van Wert	September 3, 2021	Soybean	Soybean/Corn rotation, No-till, Rye cover crop
FS47	Van Wert	September 3, 2021	Soybean	Soybean/Corn rotation, No-till, Rye cover crop
FS48	Wyandot	September 10, 2021	Soybean	Soybean/Corn rotation, No-till
FS5	Fulton	July 6, 2021	Corn	Soybean/Corn rotation
FS51	Wyandot	September 10, 2021	Soybean	Soybean/Corn rotation, No-till
FS54	Darke	September 17, 2021	Corn	Soybean/Corn rotation, No-till
FS58	Knox	October 1, 2021	Soybean	Soybean/Corn rotation
FS62	Knox	October 1, 2021	Soybean	Soybean/Corn rotation, No-till
FS63	Union	October 8, 2021	Corn	Soybean/Corn rotation, No-till
FS65	Union	October 8, 2021	Soybean	Soybean/Corn rotation, No-till

[a] Soil ID and sampling information were previously determined in Medina López et al. (2024)
[b] Data provided in sample collection form, per Medina López et al. (2024)
[c] Data not available

Supplemental Table 2. Soil macronutrients and micronutrients across the selected 18 soil samples (modified from Median Lopez et al., 2024).

Sample ID	N	S	Ca	Mg	K	Na	B	Fe	Mn	Cu	Zn	Al
	%	ppm										
FS4	84	69	2038	321	170	16	0.53	342	16	4.34	5.25	779
FS5	68	23	1244	175	107	22	0.27	154	79	4.38	4.85	555
FS11	93	32	3018	374	167	18	0.64	196	7	3.68	2.46	808
FS14	91	23	3015	387	171	16	0.59	191	9	4.32	1.94	462
FS19	74	50	1831	215	91	16	0.8	161	17	1.87	3.35	637
FS38	74	42	2878	231	137	15	0.79	116	89	2.3	2.72	303
FS39	70	41	1540	170	146	17	0.43	134	7	1.78	2.82	339
FS41	67	25	1200	223	147	13	0.25	123	83	1.96	4.26	704
FS43	74	43	3608	367	130	16	0.77	151	22	4.54	3.43	570
FS44	80	60	1557	53	222	15	0.34	153	53	1.91	3.31	597
FS47	82	32	1146	199	165	12	0.28	139	108	2.02	2.69	688
FS48	84	18	2134	60	94	14	0.43	135	60	2.36	1.28	534
FS51	82	41	1593	201	78	13	0.27	133	70	1.98	2.1	433
FS54	80	591	1939	347	188	19	0.69	194	49	5.16	7.35	512
FS58	97	56	3337	374	224	15	0.69	177	14	4.11	4.84	614
FS62	82	45	3936	280	180	14	0.7	82	66	2.9	3.34	645
FS63	72	40	1436	302	156	10	0.34	175	19	2.82	3.57	588
FS65	85	43	1631	313	167	12	0.46	177	30	1.98	1.97	546

Supplemental Table 3. Total number of reads per sequenced samples at different steps of the bioinformatic pipeline, from raw reads to number of arbuscular mycorrhizal fungal reads per sample.

Sample ID	Raw reads	Reads after trimming/QC	Reads after ASV calling	Fungal Reads	AMF Reads
FS1	20226	18172	16315	10026	13
FS11	8448	8002	7461	5034	4928
FS14	13161	10273	7192	6097	3413
FS19	23240	21267	20015	13948	13948
FS21	4801	3323	2547	2393	0
FS33	13137	11435	9958	8626	0
FS35	10892	8639	6994	5882	27
FS38	24292	22133	21975	14446	13173
FS39	13703	13330	12183	7173	6794
FS4	22068	20988	18257	6747	6274
FS41	11544	11452	10674	5552	5379
FS43	11499	11022	10880	5248	4969
FS44	11617	11008	10498	9971	9167
FS47	36234	30451	26740	24345	22490
FS48	41202	34867	29999	23120	19685
FS5	20605	19656	18059	9859	9313
FS51	4320	4092	3645	2795	2597
FS54	1927	1701	1667	1535	966
FS55	14751	11411	10600	8636	0
FS57	13221	10843	9637	9248	9
FS58	26380	24564	20755	11797	9458
FS62	33566	29768	24748	20737	19403
FS63	43008	32476	29141	10887	10405
FS65	1364	1173	962	466	402
FS67	8644	8420	8084	4830	4497
FS71	7580	7160	6093	5274	5274
FS78	6378	5356	4185	3990	21
FS80	13275	11455	10826	8309	0
AML_positive_control	14342	14050	13240	7225	6903
Negative_control	183	181	180	0	0

Supplemental Figures

Supplemental Fig 1

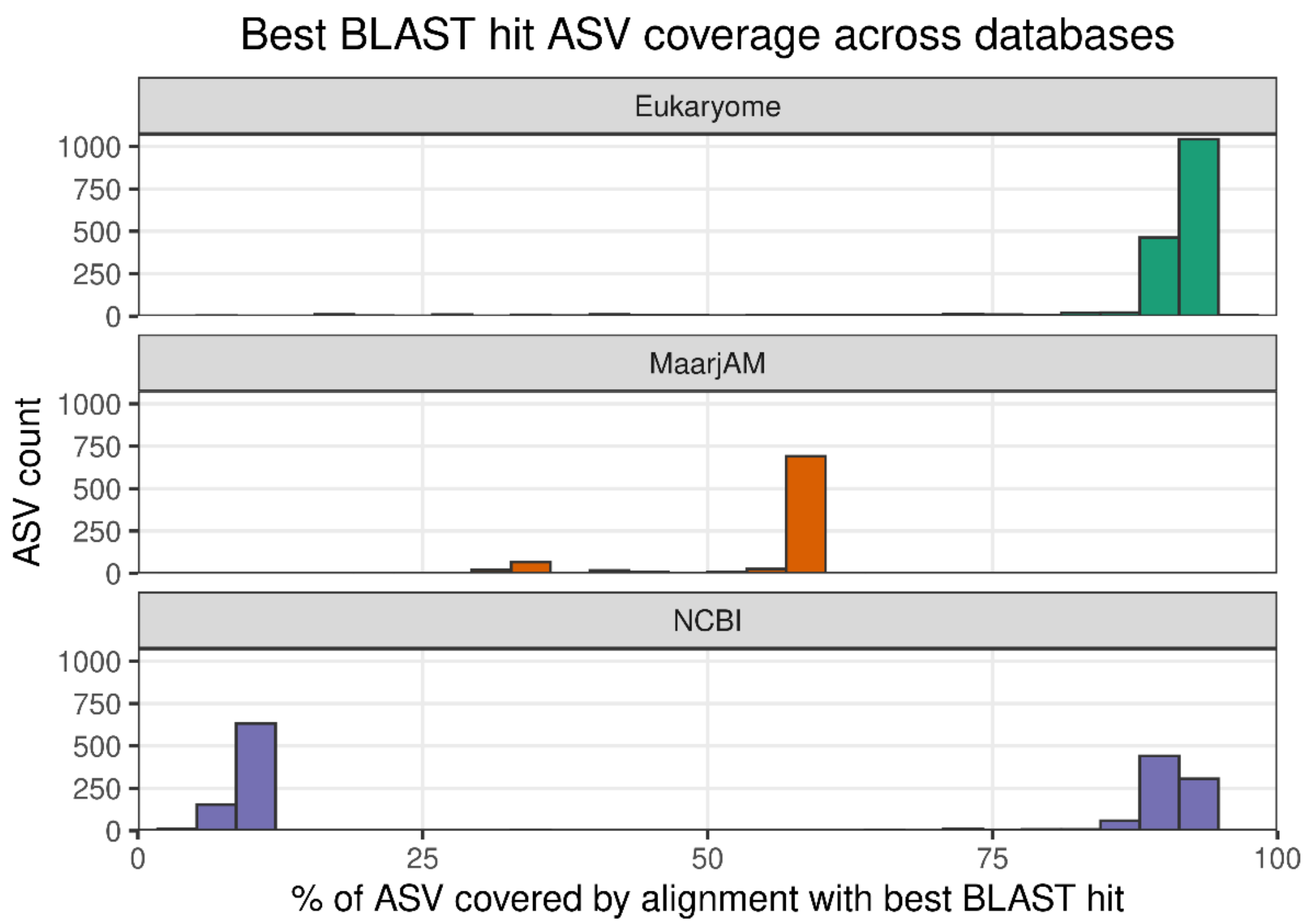

Comparative ASV coverage by best BLAST hits across taxonomic databases: NCBI 18S_fungal sequences, MaarjAM (Öpik et ak 2010), and Eukaryome databases (Tedersoo et al 2024).

Supplemental Fig 2

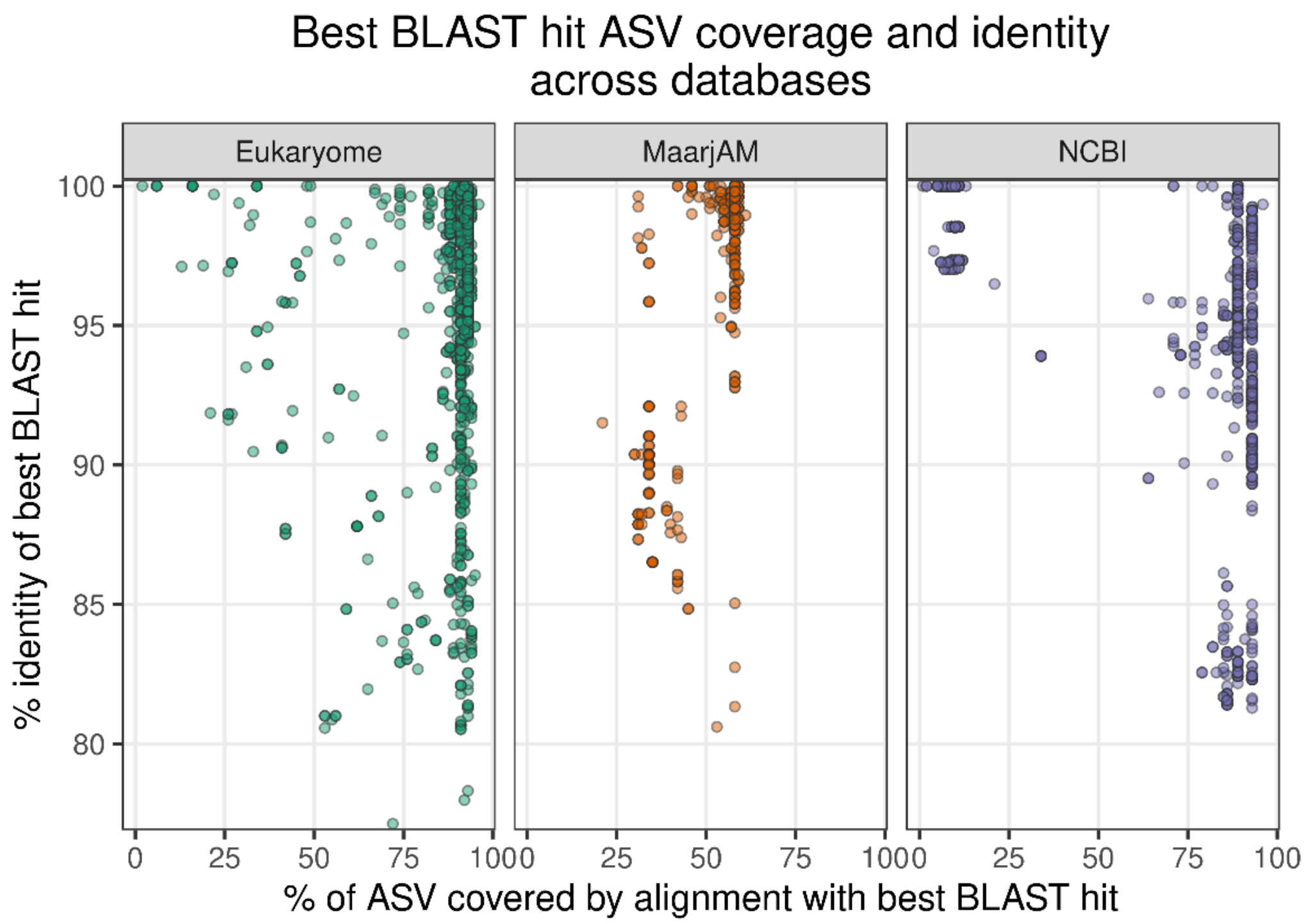

BLAST comparison of the NCBI 18S_fungal sequences, MaarjAM (Öpik et ak 2010), and Eukaryome databases (Tedersoo et al 2024). The Eukaryome database had by highest coverage and high-identity hits among the three databases and was selected for downstream analysis.

Supplemental Fig 3

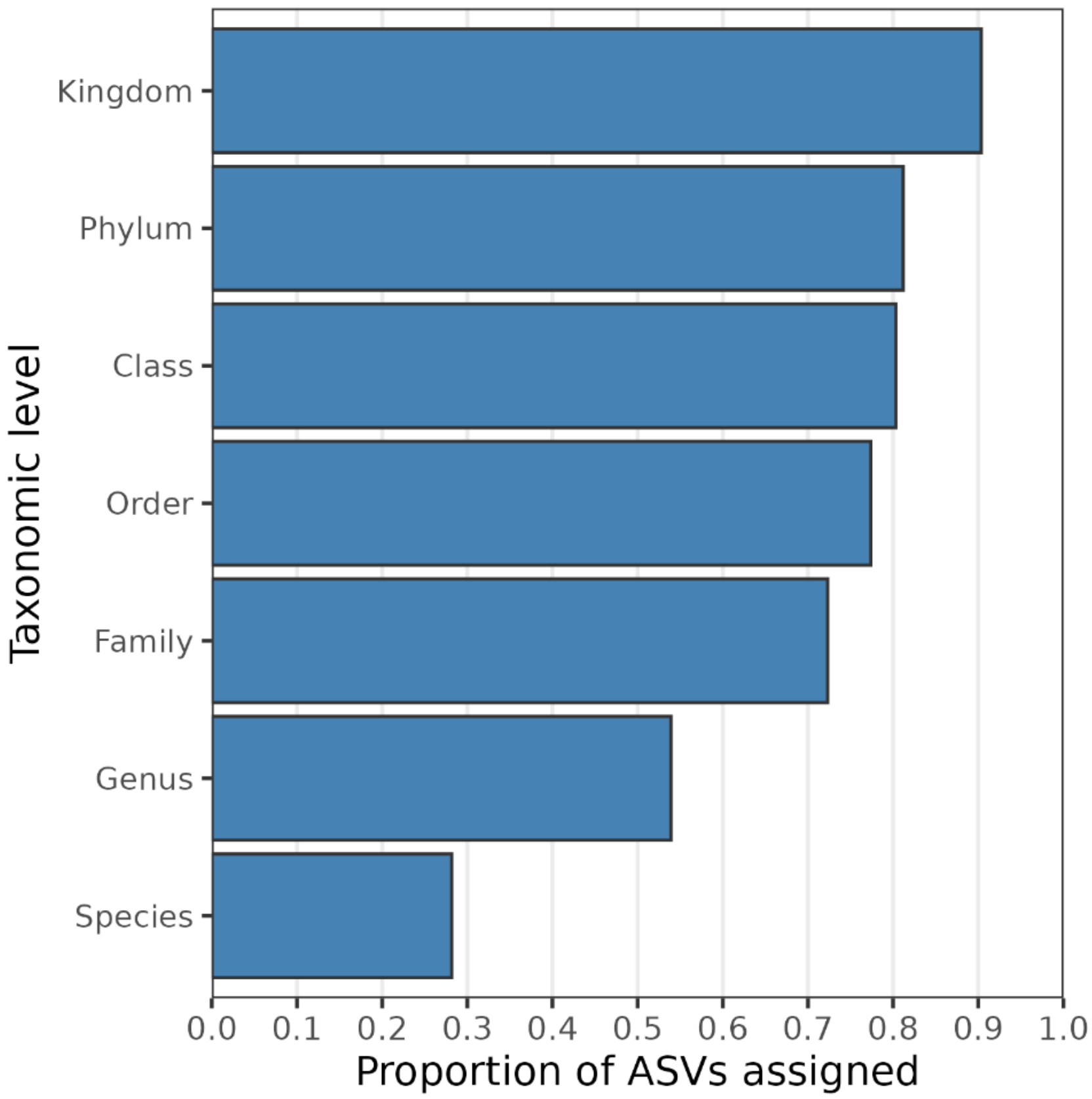

Proportion of distinct ASV assigned to a variety of taxonomic levels using the Eukaryome database (Tedersoo et al, 2024). ASV that were not assigned to Kingdom had no taxonomic assignments at all.

Supplemental Figure 4.

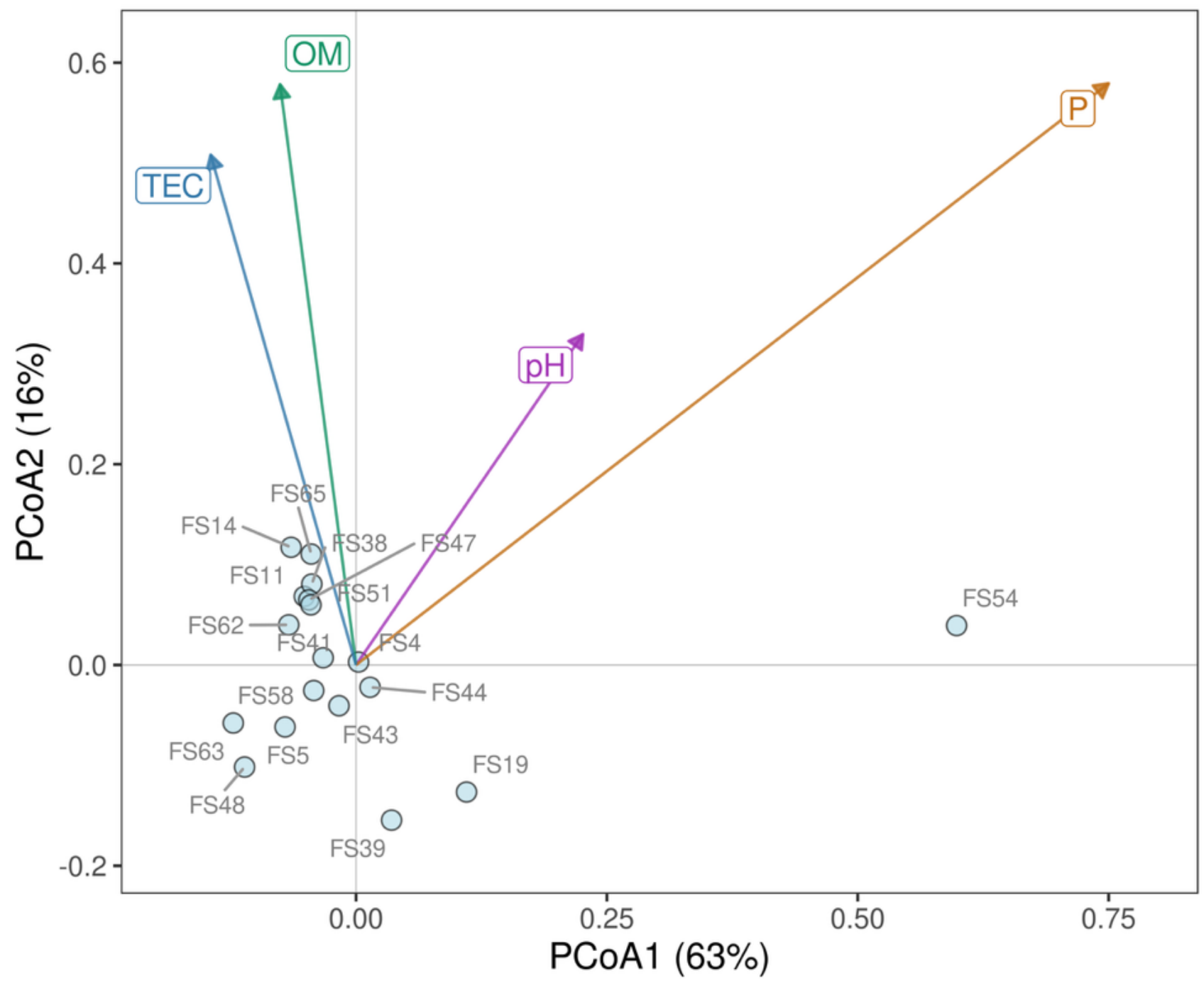

Sample-centric principal Coordinates Analysis (PCoA) of the studied fields based on AMF community composition and soil properties (pH, OM, TEC, P, colored vectors). PCA estimated based on per-sample distance matrix.

References:

Dorrance A, Martin D, Harrison K et al. Soybean Cyst Nematode. 2018.

Medina López M, Lopez-Nicora HD, Benitez Ponce MS (2024) Fungal Communities Shift with Soybean Cyst Nematode Abundance in Soils. Phytobiomes J 8:4, 568-577. https://doi.org/10.1094/pbiomes-02-24-0021-r

Leho Tedersoo, Mahdieh S Hosseyni Moghaddam, Vladimir Mikryukov, Ali Hakimzadeh, Mohammad Bahram, R Henrik Nilsson, Iryna Yatsiuk, Stefan Geisen, Arne Schwelm, Kasia Piwosz, Marko Prous, Sirje Sildever, Dominika Chmolowska, Sonja Rueckert, Pavel Skaloud, Peeter Laas, Marco Tines, Jae-Ho Jung, Ji Hye Choi, Saad Alkahtani, Sten Anslan , EUKARYOME: the rRNA gene reference database for identification of all eukaryotes, *Database*, Volume 2024, 2024, baae043, https://doi.org/10.1093/database/baae043

Öpik, M., Vanatoa, A., Vanatoa, E., Moora, M., Davison, J., Kalwij, J.M., Reier, Ü., Zobel, M. 2010. The online database MaarjAM reveals global and ecosystemic distribution patterns in arbuscular mycorrhizal fungi (Glomeromycota). New Phytologist 188: 223-241.
